# Hospital Abuse and the Working Class

**Published:** 1894-01-20

**Authors:** 


### THE INSTITUTIONAL WORKSHOP.

### HOSPITAL ADMINISTRATION.

### HOSPITAL ABUSE AND THE WORKING CLASSES.

The *Leeds Hospital Magazine* contains an account of a Conference held at the Leeds Infirmary on the alleged abuse of Leeds medical charities. Mr. F. Benson Jowett, chairman of the Infirmary Board, presided, and the Conference proceeded to consider "whether, and in what way, the number of out-patients can be reduced by dealing especially with those who, for trivial complaints, unduly occupy the time of the honorary staff of each charity, and whether any wise method can be adopted for reducing the number of patients who may be well able to pay for medical attendance outside the charities."

These questions have often been debated, and indeed a wave of consideration seems to extend through the large towns at least once in every decade. The results of such conferences, so far, have not been encouraging. The reason is not far to seek. The medical attendance is at present entirely honorary, and as a consequence those gentlemen who give many hours of each week to the gratuitous treatment of patients at hospitals and kindred institutions are held to be entitled to make a reputation by attending all and sundry who visit their consulting-rooms at the hospitals from the four corners of the city, or even of the earth. Thus at Leeds, Alderman Spark, chairman of the Workpeople's Fund, stated that out of 62,859 out-patients attending the Infirmary during the year, 34,401 came from outside Leeds, and only 28,458 patients belonged to that city.

We are not surprised to find, despite the marked success which has attended Alderman Spark's efforts so far as contributions from the working classes of Leeds are concerned, that the Leeds workpeople contributed last year £4,285, and sent 3,392 in-patients to the Infimary, whilst the outside workpeople contributed only £902, though they sent no less than 5,495, that is nearly twice as many, in-patients into the Infirmary. These figures do but illustrate the position of affairs which pretty largely prevails throughout the large cities of England. It reveals a state of affairs which tends to pauperise the people, to set the members of the medical profession by the ears, and to make many generous friends of hospitals hesitate to continue their contributions, because they hold, and we think rightly hold, that the results of the present system are calculated to do more harm than good to the mass of the people, and to the medical profession itself.

The speakers at the Leeds Conference practically left the medical aspect of the problem untouched. They stated that the chief sinners, so far as the patients were concerned, are to be found, not among the working classes, but among the lower middle class. Dr. Griffith declared that the consulting-rooms were swamped by men and women—the women being very much worse than the men—who monopolised their time and prevented the physicians from giving care to serious cases. The institution of a strict inquiry into the circumstances of each applicant for treatment was suggested, such inquiry to be conducted by the Charity Organisation Society, as a small minority thought; or by members of the Workpeople's Hospital Committee; or by issuing recommendations for distribution by members of that committee; or by fixing a wage limit for relief; or, as the balance of opinion seemed to tend, by reverting to the old recommendation forms or tickets to be issued to subscribers by the Infirmary authorities. It will be seen that these suggestions were not original, that several of them have been tried elsewhere and proved to be failures; and hence we think it may be helpful to indicate what changes in the present system seem best calculated to secure the end in view.

#### WHAT LEEDS MIGHT DO.

We take it that there is no city in the country better adapted than Leeds for the application of a new plan to the system of hospital relief. The size of its population, the number and extent of its medical charities, and the exceptional vigour and ability with which both the charities and the Workpeople's Funds are administered, all tend in favour of the introduction of wise reforms with the best prospect of success.

We gather from the speeches at the Conference that tickets or recommendations are not at present issued to subscribers by the Infirmary authorities. In other words, admission to the Infirmary is an absolutely free gift to the patients, whose medical needs form the sole passport to admission. If this be so, we strongly urge the authorities to continue this system unaltered. Free admission, however, entails the necesity of putting upon each patient the responsibility of proving to the hospital authorities, that he or she is entitled by circumstances to free treatment as an object of charity. It is not the duty of the hospital authorities to inquire into the circumstances of each patient as has been wrongly insisted upon for half a-century, but each hospital committee has a paramount obligation to insist upon each patient proving his or her title to receive free medical relief. Failing the production of such proof, free relief should be withheld or refused in all cases. The failure to grasp and apply this principle to the admission of patients to hospitals in England, as it is applied in the United States of America, has produced most, if not all, of the evils which we are now considering.

Again, it is an anachronism that the subscribing and artisan population of Leeds should provide for the medical treatment of an ever-increasing number of outsiders, who come from places which have hospitals of their own, and who contribute practically nothing to the support of the Leeds institutions. How is this predatory pillage to be prevented?

The North Staffordshire Infirmary, Stoke-upon-Trent, supplies a practical answer. There the workpeople contribute to the Infirmary on a system which enables them to ensure that only those who are entitled to the benefit shall receive treatment, whilst it enables the working classes to give a considerable sum each year, as a free gift, towards the relief expended upon the deserving sick and non-pauper poor, who are less fortunately circumstanced than the artisan class. The system is simple enough. Every subscribing workshop receives from the Infirmary books containing letters of introduction, which are issued by the foremen in each shop to the workmen or members of their family who may be ill. These recommendations, when presented at the Infirmary, entitle the applicant to medical examination and treatment as an in or out patient as the necessities of each case require. In this way the workshops, on the one hand, know precisely who receives relief at the Infirmary, and the Infirmary authorities know the nature and cost of the relief given to the patients sent by each workshop. An account is thus easily kept between the Infirmary and the workshops, and so it is not difficult to ascertain how far the contributions paid in equal or fall short of the money paid out, in relief, by the hospital.

Workpeople, as a class, have an independent spirit, and under the Stoke system, each body of workpeople in a particular shop is enabled to provide that their contributions shall be adequate, and something more, so that they may cease to be a burden on the hospitals to which they owe so much. It follows that this system would stop the outsider from preying upon the funds of any medical institution, providing an inquiry officer is employed to look after all cases which do not bring a letter of introduction from a workshop, for which latter class of cases the workshops should hold themselves responsible. We make bold to say that if this simple plan were to be adopted at Leeds, and if every patient not sent from a workshop and not producing a letter of recommendation, were asked (1) to prove his or her claim to free medical relief, or (2) to contribute something, according to their means, towards the cost of their treatment at the hospital, the whole of the present difficulties would speedily disappear.

Of course, all cases of accident and urgency should be admitted immediately as in-patients, inquiries in such cases being postponed until their friends can be communicated with, and the patient's means thus ascertained.

We earnestly commend this proposal to the consideration of the Infirmary Board, the medical profession, the workpeople and the public of Leeds, believing that it offers a wise solution to the evils which the Conference had under consideration.

### Medical Aspects of the Problem.

The medical aspects will not be found to be serious if they are approached in the right spirit. We believe, in the first instance, that the present eleemosynary system of medical treatment is demoralising to the patients, and also to the doctors. *Forward*, the monthly journal of the Birmingham Hospital Saturday Fund, speaking on behalf of the artisans of Birmingham, declares that the honorary medical staffs of our hospitals get more "plums" out of these institutions than anybody else. "The positions on the honorary staffs of these institutions are the prizes of the profession; eagerly sought for, and obtained often in the face of the keenest competition. Those who hold them are held in high repute by the community. In the hospital the medical man obtains experience which in his professional career constitutes his capital. That capital produces him a return for the services which he has gratuitously given at the hospital in the shape of increased fees."

How do the leaders of the medical profession like the sound of this word "plums," in the mouth of the working man? Is it dignified, is it necessary, nay, is it right, that all these eleemosynary services should be rendered in so unbusinesslike a way that there is danger of demoralization to the medical practitioner by making him anxious to have a large number of patients attending his out-patient rooms than his colleagues. This competition in numbers, with the view, if the truth be spoken, of a wider publicity for the individual practitioner, to enable him to secure the advantages already stated, has always seemed to us unworthy of the dignity of a great profession. In our view, the time has arrived, or nearly so, when the whole question of medical attendance should be reformed and altered. Every medical man attached to the honorary staff of a hospital or kindred institution should be paid a reasonable sum for the services he renders in that capacity. If such payment were forthcoming, the present injustices and hardships which the younger members of the honorary staffs have to face would be abolished, the numbers attending the out-patient departments of the hospitals, and the cost of hospital treatment too, would speedily be reduced to reasonable proportions, and the committees and governors would then be free to manage the hospitals on business lines, apart from any of the considerations affecting the honorary medical staff, which at present hamper every proposal in the way of reform.

We are quite confident that the dignity and honour of the medical profession would be immensely raised by such a change, and that it would at last secure justice to the great body of general practitioners, whose interests are undoubtedly seriously affected at the present time by the ever-increasing number of people who seek free relief at the hospitals. And what is even more important still, both patients and practitioners would be saved from the risks of demoralisation which undoubtedly attach to the inchoate and unbusinesslike system which at present leaves the medical attendance at British hospitals without adequate control or intelligent administration.

